# Dr. Richard Edward Sweeney

**Published:** 1917-02

**Authors:** 


**Dr. Richard Edward Sweeney,** Niagara 1898, of New York City, died at the Gouverneur Hospital Dec. 25, from burns caused by the upsetting of an oil stove.

